# Supplementary material for: Barriers to accessing high-quality cancer medicines in Cameroon. A qualitative study of the views and practices of regulators and frontline healthcare providers
Source: PLOS Glob Public Health. 2026 Jan 23;6(1):e0005370. doi: 10.1371/journal.pgph.0005370 (PMC12829877; doi:10.1371/journal.pgph.0005370)
Supplement: S1 Text — (DOCX) [file pgph.0005370.s001.docx]

**Interview guide** for the study on chemotherapy drug markets

**Qualitative interviewer's guide (pharmacists and distributors)**

**Access (availability and affordability) to anti-cancerous medication**

I.1 How would you describe access (availability + affordability) to anti-cancer drugs in the context of your activities?

I.1.1. What are the obstacles and facilitators?

I.2 What about affordability?

I.2.1. What are the obstacles and facilitators?

I.3 Which of the following molecules are still available: Cisplatin, Oxaliplatin, Methotrexate, Doxorubicin, MESNA, Leucovorin, Cyclophosphamide, Ifosfamide? Which are in high demand?

I.4 Which drugs are frequently out of stock? Why or why not?

I.5. How would you describe the source of cancer drugs?

1.6 Where do patients obtain these drugs if they are not available at the hospital? (hospital pharmacist only)

I.7. What is the speed/time of flow of chemotherapy products in your pharmacy?

I.8. Which products are generally available one month after stocking?

I.9. What are the sources of information from which you make the "forecast"? (import registers; hospital purchasing registers or national patient treatment registers; morbidity and consumption data)

I.10. How do these sources of information compare with your forecasts?

I.11. How is supply arranged (obstacles and facilities) ?

**II. Unregistered dosage forms: are other brands or dosage forms (e.g. not registered for use on your site) of these eight APIs used on your site? These may be grey market products, products purchased by patients on the private market, or products imported by private clinical sites or NGOs.**

II.1 What is the difference between registered and unregistered APIs?

II.2 Are you aware of unregistered APIs and their use? (Product representing 20% or more of the market is considered major; most widely used APIs)

II.3 What sources of information did you use to answer this question?

Can you describe the situation in a particular hospital, region or city, or in the country as a whole?

1. **Quality of anti-cancer medication**

III.1 Can you tell us about the quality of anticancer drugs in your establishment?

III.1.1. What do you know about substandard and falsified drugs?

III.1.2. Do you encounter them in your environment?

III.1.3. Does it worry you?

1. **Assessment/quality control by the regulatory authority**

IV.1. Does the national drug regulatory authority assess the quality of these products at any stage (e.g., at registration, through the PMS, or when a person files a complaint)?

IV.2 Do clinical sites carry out quality assessment activities, such as checking expiration dates, recording brand/batch numbers or visual inspection/analysis of packaging? Are these activities recorded in the patient's care record?

IV.3 What concerns do clinicians have about the quality of cancer drugs?

IV.4 What sources of information were useful in answering parts 4.1, 4.2 and 4.3?

**V. Transportion stockage:**

V.1 Are anticancer drugs shipped with special precautions (e.g. cooling devices, temperature recording devices or GPS monitoring)? Why or why not?

V.2 Are anticancer drugs stored separately from other types of drugs? Why or why not?

V.3. Are temperature-sensitive anticancer drugs always kept cold? Why or why not?

V.4. Does your answer apply to a particular hospital, a region or large city, or the whole country?

V.5. Does your information concern the public sector, the private sector or both?

V.6. Are there any differences between actual practice and regulatory requirements for transporting and storing anticancer drugs?

**VI. How the system for financing cancer drugs works**

VI.1 How does the financing system for cancer drugs work (how does the patient acquire the treatment?)?

VI.2 Is there a subsidy program for cancer drugs?

VI.3 How sustainable is the supply chain for cancer drugs?

***Thank you for your participation in this study***
