# Supplementary material for: Barriers to accessing high-quality cancer medicines in Cameroon. A qualitative study of the views and practices of regulators and frontline healthcare providers
Source: PLOS Glob Public Health. 2026 Jan 23;6(1):e0005370. doi: 10.1371/journal.pgph.0005370 (PMC12829877; doi:10.1371/journal.pgph.0005370)
Supplement: S2 Text — (DOCX) [file pgph.0005370.s002.docx]

6.3.1 Qualitative interviewer guide For Key Informants (Procurement, Private/Public pharmacy)

1. Access (Availability and affordability) of anticancer drugs
   1. How do you describe access (availability + affordability) to anticancer drugs in your practice setting?
      1. What are the barriers, Facilitators?
   2. How about their affordability?
      1. What are the barriers, Facilitators?
   3. What anticancer drugs are always available? *(Try to probe by mentioning the tracer drugs, drugs having a huge demand)*
   4. What drugs frequently stockout? Do you have any thought, why?
   5. How do you describe the source of anticancer drugs? Where did the patients get them if drugs are not available in the hospital?
   6. How fast do the chemo products move?
      1. What products are available 1 month later? *(Go back to the hospital pharmacy and if possible the community pharmacies and repeat the Pharmacy brand/batch survey–check to see whether any of the products from the previous month are still on the shelf and what new products are present)*
      2. If this gives interesting results we may want to develop this survey activity in future years.
   7. Explain how “forecasting” is made.
      1. Describe the sources for your information, for example, are you using import records provided by the drug regulator, hospital purchasing records, or national records of patient treatments? Do you use both morbidity and consumption data? If not, why?
         1. It is fine to use multiple sources?
      2. Are there any big discrepancies or gaps in information that we should be aware of?
   8. Explain how “procurement” is made, challenges and facilitators associated it?
2. Unregistered dosage forms: Are other brands or dosage forms (eg, not registered for use at your site) of these eight APIs ever used at your site? *This could include grey market products, products that patients buy on the private market, or products that are imported by private clinical sites or NGOs.*
   1. Identify any unregistered product that you feel has a major market share for its API and use case. *As a practical guide, any product that makes up 20% or more of the market is major*.
   2. What sources of information did you use to answer this question? Are you describing the situation at a particular hospital, in a region or large city, or across the entire country?
3. Treatment protocols, and or guidelines
   1. How do you describe the availability and utilization of standard treatment protocols at your practice site?
      1. Which protocol do you follow? One or more than one?
      2. Describe how general these treatment protocols are–are they used in a particular hospital, a region or large city, or the entire country?
4. Quality of anticancer medicines
   1. Can you please tell us about the quality of anticancer drugs in your setting?
      1. What do you know about sub-standard and falsified medicines?
      2. Do you encounter them in your setting?
      3. Does it make you worried?
5. Quality assessment/check by the regulatory authority
   1. Does the national drug regulator assess the quality of these products at any stage (eg, at registration, through PMS, or when someone files a complaint)?
   2. Do clinical sites conduct any quality assessment activities, such as checking expiration dates, recording brand/batch numbers, or visual inspection/packaging analysis? Are these reported as part of the record of patient care?
   3. What kind of concerns do clinicians have about the quality of chemo drugs?
   4. What information sources were useful in responding to parts a, b, and c?
6. Transportation and storage:
   1. Are chemo drugs shipped with any special precautions (eg, cold device, temperature logging devices, or GPS monitoring)?
   2. Are chemo drugs stored separately from other types of drugs?
   3. Are temperature-sensitive chemo drugs always stored at cold temperatures?
   4. Is your answer relevant to a particular hospital, a region or large city, or the entire country?
   5. Does your information include the public sector, the private sector, or both?
   6. Are there any differences between the actual practice and the regulatory requirements?
7. How is the anticancer drugs financing system (how the patient acquires the treatment? Is there anticancer drugs subsidization program? How is the supply chain sustainability of anticancer drugs)?
