# Supplementary material for: Barriers to accessing high-quality cancer medicines in Cameroon. A qualitative study of the views and practices of regulators and frontline healthcare providers
Source: PLOS Glob Public Health. 2026 Jan 23;6(1):e0005370. doi: 10.1371/journal.pgph.0005370 (PMC12829877; doi:10.1371/journal.pgph.0005370)
Supplement: Attachment — Submitted filename: PGPH-D-24-02954_review comments.docx [file pgph.0005370.s004.docx]

**Comments to the authors:**

**Abstract/introduction**

The aim as described in the abstract does not fully align with the objective described in the introduction, which focuses much more on the quality aspect. Also given the title of the work, please adjust the aim.

The term ‘supply chain’ is used, where the authors may mean the pharmaceutical value chain; the value chain also includes ‘regulation’ as a key component (see also WHO definition) where the supply chain technically does not.

It would be useful to include a definition of what the author consider as ‘quality’ in the introduction, also given the ambiguity surrounding this concept by the stakeholders interviewed.

It would be useful to provide an explanation of the standard health care structure for cancer in Cameroon. I understand that treatment is only provided in public sector hospitals? Is this government-funded or are co-payments required? For which services? Are there exceptions? Which structures are supposed to provide the medicines (if available)?
Additionally, it would be good to explain what happens if public sector pharmacies are unable to provide the require medicines (i.e. patients will buy themselves at private sector?)

**Methods**

‘Users’ are defined as oncologists and nurses. I would argue that these are in fact not the users. The patients are users. Kindly adjust wording to reflect this.

How was saturation of themes ensured or assessed?

Can the authors kindly provide the used interview guide? Additionally, what do the authors mean by ‘**originally** developed to assess the situation of anticancer medicine quality’ (line 119)? Has the interview guide previously been used in another study (if so, please provide a citation)? Did the authors conduct a pilot with the interview guide? Were any adjustments made?

Kindly provide further explanation as to how/why the two key documents were selected for review. Was there a systematic search? What were selection criteria? How did the authors ascertain that other key documents were not missed?

Can the authors clarify how and when the key documents were used in the analysis? I can imagine they were used as a starting point for getting an understanding of existing organizational and procedural structures, but this is not clear from the manuscript. Also, were these documents used to inform the interview guide or participant recruitment?
If no (semi)systematic search strategy was used, and documents were indeed used as a starting point, they authors may wish to consider whether these documents and the insights they provide should be part of the method/result sections, or rather part of the introduction as they provide background information on regulatory structures. Additionally, they don’t seem to contribute directly to identifying barriers to quality.

**Results**

Can they authors explain why there was only a single drug regulator included? Targeting seemed wider. This is in contrast to the relatively many nurses/oncologists/pharmacists included, for which thematic saturation must have been reached at an earlier point. Please provide details regarding assessing saturation in the method section. If this was not performed, this should be acknowledged as a limitation of this study.

Was there no stakeholder from the national procurement center (public sector) included? Why not?

Baseline characteristics: how were these ‘characteristics’ ascertained? Through a survey, or during the interview? How were these characteristics questioned? Again, the interview guide would be essential in understanding how these characteristics were determined.

Can the authors kindly clarify which structure is responsible for post-marketing quality control/testing? Is this the LANACOME?

Lines 227-230: Can the authors clarify how the private pharmacies depend on the public sector procurement tender?

Quote 308-312: to which stakeholder group does the program personnel belong? I cannot surmise this from the targeted groups presented in the method section.

Fig 1, please include definitions for the abbreviations (different centers) in the figure note.

Line 187: oversing = overseeing?

**Discussion**

Overall, a well-balanced discussion with clear and realistic recommendations. Perhaps the authors can also advocate more strongly/clearly for financial protection for these patients, especially in the conclusion. Affordability of medicines should not impair patient safety and treatment quality.

Lines 443-447: the authors acknowledge the lack of patients in their sample as a limitation, with which I agree. However, based on their recruitment approach, patients were never targeted in this study. Kindly clarify this: the authors chose not to recruit from this group, the lack is not due to unsuccessful recruitment of patients.

The lack of respondent from the regulatory group should be acknowledged as a limitation. The authors describe four core regulatory structures, yet only included a single regulator. Why was this? Was this due to unsuccessful recruitment? What were reasons regulators did not want to participate?

Additionally, with a single respondent, thematic saturation was surely not reached for this group.
