# Supplementary material for: Barriers to accessing high-quality cancer medicines in Cameroon. A qualitative study of the views and practices of regulators and frontline healthcare providers
Source: PLOS Glob Public Health. 2026 Jan 23;6(1):e0005370. doi: 10.1371/journal.pgph.0005370 (PMC12829877; doi:10.1371/journal.pgph.0005370)
Supplement: Attachment — Submitted filename: Response to Reviewers.docx [file pgph.0005370.s005.docx]

**Response to Reviewers**

**Reviewer** **#1**: The authors have presented a well-balanced discussion of an important aspect of access to cancer care in Cameroon, which is still too often overlooked.
Although the methodology seems generally sound and the results mostly support the authors conclusions, some critical information seems to be missing from the manuscript to fully allow me to assess this. Specifically, I would need to see the interview guide used, as well as further details regarding the recruitment strategy and saturation. The lack of included regulators is particularly concerning therein. Lastly, critical information is missing regarding the use of 'key documents'. Kindly see detailed comments attached.

**Response to reviewer 1:** Thank you very much for considering our manuscript, and for your rich review and constructive feedback. We have now added more information on the interview guide, recruitment strategy and saturation within the manuscript in the methods section, and as an attachment (Annex) to the manuscript document. Also, we have added more information to clarify our use of “additional documents”.

**Abstract/introduction**

**Reviewer #1:**

The aim, as described in the abstract, the aim does not fully align with the objective described in the introduction, which focuses much more on the quality aspect. Also, given the title of the work, please adjust the aim.

**Response**:

Thank you for this comment. We have now adjusted the aim to reflect the objective and title of this manuscript. You will find this on lines 32-34.

**Reviewer #1:**

The term ‘supply chain’ is used, where the authors may mean the pharmaceutical value chain; the value chain also includes ‘regulation’ as a key component (see also WHO definition) where the supply chain technically does not.

**Response**:

Thank you for highlighting this. Our target here is the local “supply chain,” which comprises pharmaceutical companies, wholesale distributors, pharmacies, and patients. These actors also have a “regulatory and control component” as per their specific role, such as drug quality control during production (where regulatory body (ies) step in to control standards), procurement, storage, and dispensing. For clarity and consistency within our manuscript and with current references, we have defined “supply chain” more clearly to reflect the meaning we give within this manuscript and within our context, which also includes the regulatory and controlling roles of the stakeholders. You will find this in the introduction on lines 82 - 84.

**Reviewer #1:**

It would be useful to include a definition of what the author consider as ‘quality’ in the introduction, also given the ambiguity surrounding this concept by the stakeholders interviewed.

**Response**:

We have now included a definition of “quality”, which reflects our questioning with stakeholders and the analysis within the manuscript. You will find this on Lines 71-75.

**Reviewer #1:**

It would be useful to provide an explanation of the standard health care structure for cancer in Cameroon. I understand that treatment is only provided in public sector hospitals? Is this government-funded or are co-payments required? For which services? Are there exceptions? Which structures are supposed to provide the medicines (if available)?

**Response**:

Thank you for raising this. We have added a description of the standard health care structure for cancer in Cameroon on lines 85- 95.

**Reviewer #1:**
Additionally, it would be good to explain what happens if public sector pharmacies are unable to provide the require medicines (i.e. patients will buy themselves at private sector?)

**Response**:

Thank you, this has now been included in this version of our manuscript, as you will see on lines 90-95. This is also explained within our results.

**Methods**

**Reviewer #1:**

‘Users’ are defined as oncologists and nurses. I would argue that these are in fact not the users. The patients are users. Kindly adjust wording to reflect this.

**Response**:

The word “users” has been changed to “medical personnel” to refer to oncologists and nurses. You will find this on Line 138. Thank you for highlighting this.

**Reviewer #1:**

How was saturation of themes ensured or assessed?

**Response**:

Our themes were identified from the data and reached saturation once no new themes were identified. Interview guides and study objectives were reviewed to ensure no points were left out in the code development and later theme development. This statement has been added to our analysis description within this version of our manuscript. Thank you for raising this. (Lines 168-170)

**Reviewer #1:**

Can the authors kindly provide the used interview guide? Additionally, what do the authors mean by ‘**originally** developed to assess the situation of anticancer medicine quality’ (line 119)? Has the interview guide previously been used in another study (if so, please provide a citation)? Did the authors conduct a pilot with the interview guide? Were any adjustments made?

**Response**:

The interview guide has now been added to the manuscript as “supporting information.” A pilot study was conducted with the interview guides to test their usability in terms of understanding, the length of time needed with participants, and any adjustments were integrated. We have added this within the methods for clarity (Lines 155-156). The guide used for this study was adapted from what was developed and used in a study in Ethiopia. Like our findings, the results from their research have been submitted for publication in another journal, and this is still under review.

**Reviewer #1:**

Kindly provide further explanation as to how/why the two key documents were selected for review. Was there a systematic search? What were selection criteria? How did the authors ascertain that other key documents were not missed?

**Response**:

Thank you for raising this. Purposively, we identified and accessed more than two documents based on the existing scope for National Cancer strategies and reporting; the sources listed are websites in charge of anticancer medications and treatment and care, and within these sites, there are several documents and reports. We have made this clearer now, so as not to limit our document review to two papers only. Also, other than the published sites or documents, we considered unpublished national strategies and reports, which we have added to our references now. You will find these on Lines 157-161 (and listed amongst the references)

**Reviewer #1:**

Can the authors clarify how and when the key documents were used in the analysis? I can imagine they were used as a starting point for getting an understanding of existing organizational and procedural structures, but this is not clear from the manuscript. Also, were these documents used to inform the interview guide or participant recruitment?
**Response**:

The review added depth to most of the participant responses and assisted in verifying the validity of some statements as part of data triangulation. A statement to show this has been added on Lines 160-163

**Reviewer #1:**

If no (semi)systematic search strategy was used, and documents were indeed used as a starting point, they authors may wish to consider whether these documents and the insights they provide should be part of the method/result sections, or rather part of the introduction as they provide background information on regulatory structures. Additionally, they don’t seem to contribute directly to identifying barriers to quality.

**Response**:

Thank you for highlighting this. The document review was a key step with documents purposively selected, and other than identifying regulators and their different tasks and roles during our study, they add depth to most of the participant responses, assist in verifying the validity of statements, and assist in our discussion. We would prefer, if you permit, to include this as a result within our study. We have added a statement to support this within the methods, as you will find on Lines 157-162

**Results**

**Reviewer #1:**

Can they authors explain why there was only a single drug regulator included? Targeting seemed wider. This is in contrast to the relatively many nurses/oncologists/pharmacists included, for which thematic saturation must have been reached at an earlier point. Please provide details regarding assessing saturation in the method section. If this was not performed, this should be acknowledged as a limitation of this study.

**Response**:

As mentioned within our manuscript, the four regulatory groups are found within one main body (MINSANTE) and each has specific tasks such as verification and distribution of licenses, etc. The main body (ies) involved in technical drug quality control are the DMLEP and the LANACOME, which both have complementary roles and tasks (if not too similar) with similar staff cutting across as they are all part of the “MINSANTE”. Our regulator participant thus represents both bodies, and, in their role, we found them sufficient for questioning, in addition to reports from MINSANTE.

Thank you for highlighting this, and we have added more information to clarify this within this version of our manuscript on Lines 130-134, 184, 204-205.; and further explained from lines 205-225. Still, we have mentioned the use of only one regulator as a limit in our work (line 472). Also, the statement on saturation has been added within the methods on Lines 122 – 127.

**Reviewer #1:**

Was there no stakeholder from the national procurement center (public sector) included? Why not?

Baseline characteristics: how were these ‘characteristics’ ascertained? Through a survey, or during the interview? How were these characteristics questioned? Again, the interview guide would be essential in understanding how these characteristics were determined.

**Response**:

A stakeholder from the national procurement center (public sector) was included. We described them as program personnel/procurement, to keep their identity. The National Cancer Program and the National Procurement Center have the same role of procuring anticancer medications. They are quoted on line 343

The baseline characteristics were ascertained during our interviews.

**Reviewer #1:**

Can the authors kindly clarify which structure is responsible for post-marketing quality control/testing? Is this the LANACOME?

**Response**:

Yes, this is the LANACOME (though the DMLEP also monitors this). We have clarified this again on lines 133; 210-212

**Reviewer #1:**

Lines 227-230: Can the authors clarify how the private pharmacies depend on the public sector procurement tender?

**Response**:

Thank you. We have added a paragraph which clarifies this procedure (Lines 278 – 289). When the National procurement agency faces treasury changes, this limits their ability to pay manufacturers before importing the medications. Thus, the government issues tenders, which private pharmacies can compete for. In the instance which they obtain a “good” in the evaluation process [notably the technical (70%) and financial position (30%)], they can be/or are awarded the contract to import the medications and supply them to the government via the National procurement agency, which then distributes the medicines to the cancer treatment centers.

**Reviewer #1:**

Quote 308-312: to which stakeholder group does the program personnel belong? I cannot surmise this from the targeted groups presented in the method section.

**Response**:

Thank you. We have clarified the respondent's description. The program personnel fall under procurement, as this is one of the central roles of the program. We have added “procurement” to their description, as you will find on lines 347-348.

**Reviewer #1:**

Fig 1, please include definitions for the abbreviations (different centers) in the figure note.

**Response**:

This has now been adjusted (Lines 229 – 232)

**Reviewer #1:**

Line 187: oversing = overseeing?

**Response**:

Thank you, this has now been corrected. (now line 213)

**Discussion**

**Reviewer #1:**

Overall, a well-balanced discussion with clear and realistic recommendations. Perhaps the authors can also advocate more strongly/clearly for financial protection for these patients, especially in the conclusion. Affordability of medicines should not impair patient safety and treatment quality.

**Response**:

Thank you. We have now included a phrase in the conclusion which advocates for financial protection for patients. (Lines 528-531, and 539)

**Reviewer #1:**

Lines 443-447: The authors acknowledge the lack of patients in their sample as a limitation, with which I agree. However, based on their recruitment approach, this study never targeted patients. Kindly clarify this: the authors chose not to recruit from this group; the lack is not due to unsuccessful recruitment of patients.

**Response**:

Thank you, we have clarified this as you will see on Lines 472-481

**Reviewer #1:**

The lack of respondent from the regulatory group should be acknowledged as a limitation. The authors describe four core regulatory structures, yet only included a single regulator. Why was this? Was this due to unsuccessful recruitment? What were reasons regulators did not want to participate?

Additionally, with a single respondent, thematic saturation was surely not reached for this group.

**Response**:

As mentioned within our manuscript, the four regulatory groups are found within one main body (MINSANTE) and each has specific tasks such as verification and distribution of licenses, etc. The main body (ies) involved in technical drug quality control are the DMLEP and the LANACOME, which both have complementary roles and tasks (if not too similar) with similar staff cutting across as they are all part of the MINSANTE. Our regulator participant thus represents both bodies and, in their role, we found them sufficient for questioning, in addition to reports from MINSANTE. Still, we have mentioned this as a limit in our work (line 472).

Thank you for highlighting this, and we have added more information to clarify this within this version of our manuscript on Lines 130-134; 184; 204-205.; and further explained from lines 205-225.

**Reviewer #2:**

Very good introduction. Among the reasons for poorer outcomes in LMICs (lines 65-69), I would cite delayed access to medicines (recent Health Affairs paper by Jouni Kuha).

**Response**:

Thank you for this rich resource. We have now added this on Line 69.

**Reviewer #2:**

In the introduction I would present further information about the factors that may impact the quality of pharmaceutical care in a country like Cameroon (ranging from regulatory issues to adequate fiscal space etc.). This would provide a useful background information / framing for interpreting the results section and the myriad factors associated with the quality of care. It was otherwise a bit difficult to follow the results section, where the authors jumped from one issue (like administrative procedures and governance structures) to another (like procurement processes). An overarching framework for how this all fits together would be useful.

**Response**:

Thank you for flagging this. We have added a paragraph in the introduction, which provides brief overview of these factors. You will find this in the introduction from lines 76- 81.

**Reviewer #2:**

The methods would benefit from further description / details. The authors should include the semi-structured interview guide so that readers can better understand the methods and interpret the findings. How was the guide developed? Was it piloted? The lack of adequate detail made it very difficult for the reader to follow the Results and understand the nature of the conversations between the researchers and participants.

**Response**:

Thank you, we have now added more details to the methods section on the guide and pilot of the tools. You will find this on Lines 155-156, and also a copy of the guide added as “Supporting Information”

**Reviewer #2:**

Why was there such an uneven number of participants from the various groups (very few regulatory officials)? It’s unclear why the authors describe the results related to quality / affordability at the top of the Results; those results seem like they would fit better in the respective sections.

**Response**:

Thank you for raising this. As mentioned within our manuscript, the four regulatory groups are found within one main body (MINSANTE) and each have specific tasks such as verification and distribution of licenses, etc. The main body(ies) involved in technical drug quality control are the DMLEP and the LANACOME, which both have complementary roles and tasks (if not to similar) with similar staff cutting across as they are all part of the MINSANTE. Our regulator participant thus represents both bodies and, in their role, we found them sufficient for questioning, in addition to reports from MINSANTE.

We have now added more information to clarify this within this version of our manuscript on Lines 130-134; 184; 204-205.; and further explained from lines 205-225. Still we have mentioned the use of only one regulator as a limit in our work (line 472).

Other than this, the key informants were selected purposively, and till saturation for each role (except for regulators, as we have explained the reason above, and within the manuscript)

In relation to our highlighting “Quality/Affordibility” amongst the summary of our findings at the start of the results section, we found quality to be directly related to affordability and vice-versa at some point, and highlight these as key findings in the summary. Further amongst the results, we explained this better as seen from Lines 315-336

**Reviewer #2:**

The description of the governance structure is not really a result – this could be described in the Introduction. Any participant comments about the strengths / weaknesses of this structure / these organizations should remain in the Results (the participants’ comments about these structures). The purely descriptive components / Figure can go in the Intro.

**Response**:

Thank you for highlighting this. The document review was a key step with documents purposively selected, and other than identifying regulators and their different tasks and roles during our study, they add depth to most of the participant responses, and assist in verifying the validity of statements, and as well, assist in our discussion. We prefer, you would permit, to let this as a result within our study. We have added a statement to support this within the methods as you will find on Lines 157-162

**Reviewer #2:**

Discussion -- the authors should not refer to their own study as “seminal” – this is usually something judged by readers. I think the authors are trying to make the point that this is the first study to explore these issues in Cameroon, which is something useful to state.

**Response**:

Thank you. This has been integrated and we have taken out the word “seminal” (Line 413)
