# Supplementary material for: Barriers to accessing high-quality cancer medicines in Cameroon. A qualitative study of the views and practices of regulators and frontline healthcare providers
Source: PLOS Glob Public Health. 2026 Jan 23;6(1):e0005370. doi: 10.1371/journal.pgph.0005370 (PMC12829877; doi:10.1371/journal.pgph.0005370)
Supplement: Attachment — Submitted filename: PGPH-D-24-02954_R1_review comments.docx [file pgph.0005370.s006.docx]

**Comments to the authors:**

Firstly, thank you for providing the interview guide. I note that, although the title of the manuscript highlights the “perceptions” of stakeholders, the guide mostly asks about their practices rather than opinions or experiences. The result section, in turn, seems to report both. I suggest the authors clearly emphasize they assessed both practices and perspectives in their title, abstract and introduction/objectives. Additionally, I believe this approach should also be acknowledged in the discussion section: the interview guide included a limited number of questions on the participant’s perceptions (‘how do you feel about…’, ‘how do you experience….’, etc.); instead, their perceptions where teased out of their answers on factual questions.

Along the same lines, although the authors mention that the baseline characteristics were ascertained during the interviews, I cannot match them with questions in the interview guide. The authors should be careful with reporting qualitative data as quantitative data (i.e. reporting 60% acknowledged quality was a challenge, when the respondents were not literally asked this).

The authors could also shortly add to the method section the main themes that were covered in the interview guide, and refer to the supplementary material in the main text.

The explanation of the pilot could benefit from a bit more detail. Was the guide tested in Cameroon or in Ethiopia? On how many participants? Were there results used in the final manuscript? Etc.

Saturation is now addressed in several places throughout the manuscript. It is still not fully clear to me whether saturation was assessed based on meeting the pre-established recruitment targets (if there were any, please specify), or based on thematic saturation. I would also suggest that the information now provided in lines 127-132 is instead moved to ‘analysis’ (under methods), and the limitation section in the discussion. Lastly, although the limited number of regulators is acknowledged as a limitation, it could benefit from a bit more explanation.

I understand now how the key documents were used in the analysis, and that a broader search was used to identify documents. The authors could add what selection criteria were used for inclusion. Also, were the documents screened before interviewing, or after? If first, please adjust the structure of the manuscript accordingly.

Please be aware that MINSANTE is not common terminology for me, and perhaps other readers as well. I now understand it is the ministry of health, but this could be explicitly mentioned somewhere.

The manuscript would benefit from a rigorous read-through. The result section and discussion read well, but particularly the introduction and method section contain some mistakes and are not always clearly written.
