# Supplementary material for: Barriers to accessing high-quality cancer medicines in Cameroon. A qualitative study of the views and practices of regulators and frontline healthcare providers
Source: PLOS Glob Public Health. 2026 Jan 23;6(1):e0005370. doi: 10.1371/journal.pgph.0005370 (PMC12829877; doi:10.1371/journal.pgph.0005370)
Supplement: Attachment — Submitted filename: Response_to_Reviewers_auresp_2.docx [file pgph.0005370.s007.docx]

**Editor’s Comments to Authors**

The authors have done a good job revising their manuscript. In addition to responding to the reviewer’s comments, please also address the following issues.

Please change the title to “Barriers to accessing high-quality cancer medicines in Cameroon: A qualitative study of the views and practices of regulators and front-line health care providers”

- **Response:** Thank you for this proposition. We have now changed our title to read accordingly, and find this is more representative of our methodology and results.

Please use the term “front-line” health care providers throughout the paper.

- **Response:** We have now used the term front-line, through-out the manuscript as you will find on lines 137, 212, 592, 608.

I just want to confirm that all the references in lines 75-83 relate to Cameroon specifically. Why did the authors bring up an example from Ethiopia on lines 84-85? I would consider removing.

- **Response:** Thank you for raising this, we have now removed the reference and phrase which highlights Ethiopian experience from this paragraph, leaving only details which focus on Cameroon (Line 95-96)

Avoid vague statements like “(which is more often than not)” (line 97). Delete, or replace with exact % to make clear the scale of the problem (I think these might be the percentages shown in the next sentence).

- **Response:** We have taken off the vague statements, and preferably left the following sentence as it clearly shows the source of anticancer medications in Cameroon (Line 115-119)

Please avoid the use of causal language (for example, “these weaknesses … encouraged the proliferation of SF medicines” in the abstract). Also avoid referring to “our setting”; instead, state “Cameroon” or refer to “the setting” (or “this setting” if it’s already been described)

- **Response:** The use of causal languages have been reviewed through-out our manuscript and taken out or sentences rephrased to be less causal. Examples of these can be found on Lines 53-54, 78, 557-561. Similarly, we have clearly refered to “Cameroon” and taken out the term “in our setting” through-out the manuscript. Thank you for raising these points.

Avoid the use of bullets in the methods / results. Turn these parts into paragraphs.

- **Response:** The bullets within the methods have been removed and replaced with paragraphs as requested (Lines 163-172)

Line 157 – an average would be a single number, so do you mean “lasted between 35 and 60 minutes”?

- **Response:** Thank you, we have replaced the average here with “lasted between 35and 60 minutes” and this represents better our interview lengths.

Line 197 - at the start of the Results, repeat the total number of interviewees (57)

- **Response:** The total number of interviewees have been repeated at the start of the results (Line 272), and this makes the paragraph read better. Thank you.

Lines 201-207 (and rest of Results). Provide sample size when giving % (over 70% refers to what number out of 57? Or what subset if you’re just referring to the fraction of pharmacists?). Also note that the sentence would read better if you wrote “Over 70% (X/Y) of pharmacists did not consider packaging irregularities as a quality concern.”).

- **Response:** We have now represented the percentage with the sample size, rephrasing the sentence to read “Over 70% (19/25) of pharmacists did not consider packaging irregularities as a quality concern.” (Line 282).

Lines 475-480 – did study 38-40 show causal effects? Avoid overstating the impact of these approaches unless there is conclusive evidence. Instead refer to these CHAI initiatives as examples of efforts to improve access to cancer drugs in Cameroon (not as the “primary drivers” of improvements, which is a strong claim). Avoid language like “remarkable results of improved availability and affordability … and patient outcomes” – instead summarize objective findings from studies and let readers interpret whether these results are “remarkable” or not.

- **Response:** Thank you, we have rephrased this sentence to not overstate the impact of these approaches as the conclusive evidence is only inferred as of now. We have rephrased the sentences on lines 556-561.

Please also review the paper carefully for grammar, spelling, and overall clarity. The following are some examples; this list is not exhaustive. I would carefully copy-edit the entire manuscript.

Line 34 - “in order” is misspelled as “inorder”

- **Response:** Thank you this has been corrected (line 42)

Line 35 – delete the word “some” (same thing in line 126 of the methods)

- **Response:** This has now been deleted in both places (lines 44, 230)

Line 38 – delete the word “emanating”

- **Response:** This has been deleted (47)

Line 39 – delete “which enabled the identification of key themes.”

- **Response:** This has now been deleted, thank you for highlighting this as the sentence reads better.

Line 63 - “10million” is missing a space

- **Response:** Thank you, this has been corrected (Line 76)

Line 65 – change “has been attributed to” to “may reflect”

- **Response**: Thank you, this has been changed (Line 78)

Line 70 - “showing approximately 30% only of patients…” should read “that only approximately 30% of patients …”

- **Response**: This has now been replaced (Line 83-84)), Thank you.

Line 125 – change “as well, a documentary review of some …” to “We also reviewed key administrative reports to provide key background information, triangulate data, and substantiate claims made by interviewees.” (if this is what you meant).

Response: Thank you, this is actually what we meant, and this have changed the sentence accordingly (Line 151-153)

Line 125 – “personnel within the national cancer treatment scene” is somewhat awkward phrasing (especially the “scene” part). Perhaps substitute with something like “stakeholders involved in national cancer treatment efforts”

- **Response**: We have rephrased the sentence using your suggestion, thank you (150-151)

Line 127-128, the sentence has an incorrect verb construction / comma splice: “Saturation was arrived at once, information received was similar, amongst similar role participants”. Also, this same point is made on lines 173174. I would not repeat points. Please only describe saturation in one place.

- **Response**: We have now reconstructed this sentence to read: “Saturation was arrived at once information received was similar amongst participant groups. In the case of regulatory bodies, their objectives and roles were similar, so we focused on talking only to regulators involved directly in anticancer post-marketing drug testing (this limited the number of respondents required amongst regulators)”. The description of data saturation as well, as now been limited only to one sentence in this section (Line 153-156)

Line 134-136 – this sentence is not grammatically correct

- **Response**: The whole sentence has now been rephrased with the right verb constructions and comma splice (153-156). Thank you for highlighting this.

Line 445 – split into two sentences (“supply chain; over 70% of those in private pharmacies interviewed in this study failed to acknowledge …” – or something like that).

- **Response**: The sentence has been split, and rephrased (534-536). Thank you.

Line 448 – “It’s” should be “Its”

- **Response**: This has now been corrected (line 529)

Line 462 – delete “In this same line”

- **Response**: This has been deleted (now line 546)

Line 462-465 – this is a run-on sentence. Rewrite.

- **Response**: The sentence has now been re-written and reads better (lines 546-548). Thank you.

Lines 499-500 – delete “This will have been … to the study” (the sentence just before that is very long – break in two).

- Response: We have now deleted this sentence as requested, the other broken into two, which reads better. (lines 594-597)

Again, this is not an exhaustive list at all. There were many sentences that I found difficult to follow. I would suggest revisiting the full text with a view to improving flow and readability.

- **Response**: Thank you very much. We have now taken the time to read again and revise the manuscript for grammar, spelling, and over all clarity.

**R1 Comments to the authors:**

Firstly, thank you for providing the interview guide. I note that, although the title of the manuscript highlights the “perceptions” of stakeholders, the guide mostly asks about their practices rather than opinions or experiences. The result section, in turn, seems to report both. I suggest the authors clearly emphasize they assessed both practices and perspectives in their title, abstract and introduction/objectives. Additionally, I believe this approach should also be acknowledged in the discussion section: the interview guide included a limited number of questions on the participant’s perceptions (‘how do you feel about…’, ‘how do you experience….’, etc.); instead, their perceptions where teased out of their answers on factual questions.

- **Response:** Thank you very much. We have taken note of this and have now clearly emphasized that our methodology and results examine/show practices and perceptions of frontline health workers. We further emphasized our limited direct questioning on perceptions in the Methodology. You will find these adjustments in the Title; Abstract (lines 39-40); Introduction (lines 36); Methods (lines 211 to 214)

Along the same lines, although the authors mention that the baseline characteristics were ascertained during the interviews, I cannot match them with questions in the interview guide. The authors should be careful with reporting qualitative data as quantitative data (i.e. reporting 60% acknowledged quality was a challenge, when the respondents were not literally asked this).

- **Response:** Baseline characteristics were gotten from both our document review and interviews. Most of these, like perceptions were collected from responses during the open-ended interviews. We have adjusted the text in our methodology to show that the document review went on alongside interviewing (lines 229-230). The interview guide though did not have direct questioning on quality challenges, guided the discussions towards this, and the responses had elements indicating their perceptions. We have adjusted the wording in this instance, and now say “most of study respondents”, to limit reporting our qualitative data as quantitative. You will find this on lines 276-277.

The authors could also shortly add to the method section the main themes that were covered in the interview guide, and refer to the supplementary material in the main text.

- **Response:** Thank you for highlighting this. On Lines 214-216, in addiiton to the main topics probed, we have guided the reader to interview guide.

The explanation of the pilot could benefit from a bit more detail. Was the guide tested in Cameroon or in Ethiopia? On how many participants? Were there results used in the final manuscript? Etc.

- **Response:** We have now added more details on the pilot testing site, and also its use within the manuscript. You will find this on lines 215-217.

Saturation is now addressed in several places throughout the manuscript. It is still not fully clear to me whether saturation was assessed based on meeting the pre-established recruitment targets (if there were any, please specify), or based on thematic saturation. I would also suggest that the information now provided in lines 127-132 is instead moved to ‘analysis’ (under methods), and the limitation section in the discussion. Lastly, although the limited number of regulators is acknowledged as a limitation, it could benefit from a bit more explanation.

- **Response:** Saturation was firstly arrived at during data collection with participant interviewing. When information received was similar amongst similar role participants, we did not move further to interview any more respondents of the same group. This we have explained on lines 129-134. Considering this was during data collection and not formal analysis, if you will permit, we prefer to leave this information within the methods description. In the analysis section, we have mentioned as well, thematic saturation was arrived at during theme developement during formal analysis (line 179-180).

I understand now how the key documents were used in the analysis, and that a broader search was used to identify documents. The authors could add what selection criteria were used for inclusion. Also, were the documents screened before interviewing, or after? If first, please adjust the structure of the manuscript accordingly.

- **Response:** Documents were reviewed alongside interviewing. These were complementary activities. We have added a sentence to clarify this on lines 229-231.

Please be aware that MINSANTE is not common terminology for me, and perhaps other readers as well. I now understand it is the ministry of health, but this could be explicitly mentioned somewhere.

- **Response:** Thank you, we have reviewed this. On line 163, we have clearly defined MINSANTE as Cameroon Ministry of Public Health.

The manuscript would benefit from a rigorous read-through. The result section and discussion read well, but particularly the introduction and method section contain some mistakes and are not always clearly written.

- **Response:** We have now carefully reviewed this version of our manuscript for clarity and to improve flow and readability. Thank you for bringing this out.
