# Supplementary material for: Barriers to accessing high-quality cancer medicines in Cameroon. A qualitative study of the views and practices of regulators and frontline healthcare providers
Source: PLOS Glob Public Health. 2026 Jan 23;6(1):e0005370. doi: 10.1371/journal.pgph.0005370 (PMC12829877; doi:10.1371/journal.pgph.0005370)
Supplement: Attachment — Submitted filename: Response to Reviewers-2.docx [file pgph.0005370.s008.docx]

**Editor’s Comments to Authors**

The authors have done a good job revising the manuscript. Could they please address the following minor issues.

There is a typo in affiliation #2 (Institute). The affiliations don’t follow a consistent format.- #5 lists the state (NC), whereas #9 lists the city (Notre Dame). Also please list departments before university names (#3-6 and #8-9). Please check all author names and affiliations again carefully for typos.

**Response:** Thank you for highlighting this. We have now corrected the typo in “Institute” and have consistently used the city for every Institution. Also, we have now placed all departments before university name. Author names and institutions have been verified (lines 10 – 18)

Line 69- change “…disproportionate access to quality medicines [11], delayed access to medicines [12], and high circulation of substandard and falsified anticancer medications in LMIC markets” to “…inadequate access to quality assured-assured medicines [11-13]”.

**Response:** We have now changed this phrase as you will find on lines 81. Thank you.

Avoid acronyms as this makes the text very difficult to read. Spell out all the acronyms that are not used throughout the manuscript (such as SF, PPE, IDIs, some of the government bodies – instead of using IDIs with every quote, is it possible to use some other convention to make clear that these quotes are from respondents? for example, perhaps the authors could assign #s to the respondents, e.g., “Regulator #3, “Oncologist #7”, etc.)

**Response:** Thank you, we have now taken out the acronym “IDI” everywhere within the manuscript. We have identified the respondents with #s. Also, we have taken out acronyms that are not used throughout the text (like MINSANTE and DMLEP) and specified “SF” everywhere it is used. (Lines 203, 264, and throughout the manuscript)

Lines 136-147 – Please avoid lists in the main text. Either convert to a narrative format or list the stakeholders in a Box. For example, “The respondents fell into three main groups: (1) regulators and quality-control officials; (2) procurement personnel; and (3)health-care personnel. The regulatory group comprised x, y and z. The procurement group consisted of …” Something along those lines.

**Response: Thank you for this.** We have now put the phrase in a narrative form. (Lines 158-167)

Avoid bullet points in the results (lines 250-256)- please convert into paragraph format. Also, please note that you are missing the end of the sentence on lines 255-256 (“…which in turn result in)

**Response**: Thank you, we have taken off the bullet points (lines 295-302), and the incomplete sentence has been resolved (line 302).
